# Multicomponent training versus elastic-band training for improving global cognition and executive function in older women: a randomized controlled trial

**DOI:** 10.3389/fnagi.2026.1911384

**Published:** 2026-09-30

**Authors:** Edgar Vásquez-Carrasco, Pablo Aravena-Sagardia, Mauricio Barramuño-Medina, Sebastián Valenzuela-Rosel, Victor Alarcón-Zambrano, Jordan Hernandez-Martinez, Roberto López-Andaur, Cristian Sandoval-Vásquez, Braulio Henrique Magnani Branco, Pablo Valdés-Badilla

**Affiliations:** 1 School of Occupational Therapy, Faculty of Psychology, Universidad de Talca, Talca, Chile; 2 Centro de Investigación en Ciencias Cognitivas, Facultad de Psicología, Universidad de Talca, Talca, Chile; 3 VITALIS Longevity Center, Universidad de Talca, Talca, Chile; 4 Postgraduate Program in Health Promotion, Cesumar University, Maringá, Paraná, Brazil; 5 Physical Education Pedagogy, Faculty of Education. Universidad Autónoma de Chile, Temuco, Chile; 6 Kinesiology Program, Faculty of Health Sciences, Universidad Autónoma de Chile, Temuco, Chile; 7 Department of Psychology, Faculty of Health Sciences, Universidad Católica de Temuco, Temuco, Chile; 8 Department of Physical Activity Sciences, Universidad de Los Lagos, Osorno, Chile; 9 Department of Education, Faculty of Humanities, Universidad de la Serena, La Serena, Chile; 10 Exercise and Rehabilitation Sciences Institute, School of Occupational Therapy, Faculty of Rehabilitation Sciences, Universidad Andres Bello, Santiago, Chile; 11 Escuela de Tecnología Médica, Facultad de Salud, Universidad Santo Tomás, Los Carreras, Osorno, Chile; 12 Departamento de Medicina Interna, Facultad de Medicina, Universidad de La Frontera, Temuco, Chile; 13 Department of Physical Activity Sciences, Faculty of Education Sciences, Universidad Católica del Maule, Talca, Chile; 14 Sports Coach Career, School of Education, Universidad Viña del Mar, Chile

**Keywords:** aged, exercise therapy, female, neuropsychological tests, resistance training

## Abstract

**Objective:**

To compare the effects of multicomponent training (MCT) and elastic-band training (EBT) on global cognition and executive function in older women.

**Methods:**

This single-blind, parallel-group randomized controlled trial was conducted over 16 weeks and included two supervised 60-min sessions per week. Twenty-six older women were randomly assigned to either the MCT group (*n* = 13) or the EBT group (*n* = 13). Cognitive outcomes included global cognition assessed with the Montreal Cognitive Assessment (MoCA), processing speed and visual attention assessed with the Trail Making Test Part A (TMT-A), and executive function assessed with the Trail Making Test Part B (TMT-B). Linear mixed-effects models were used to evaluate group, time, and group × time effects while adjusting for age and education. TMT-B was predefined as the primary executive function outcome.

**Results:**

A significant group × time interaction was observed for TMT-B performance (*p* = 0.019). However, neither the within-group nor between-group *post hoc* comparisons remained significant after false discovery rate (FDR) correction, and the interaction did not remain significant when FDR correction was conservatively applied across all model-level interactions. No significant group × time interactions were observed for MoCA (*p* = 0.289) or TMT-A (*p* = 0.214).

**Conclusion:**

Although the primary model suggested different temporal trajectories in TMT-B performance, the nonsignificant adjusted *post hoc* comparisons and the conservative sensitivity analysis preclude any conclusion that MCT was superior to EBT. Neither intervention produced significant changes in global cognitive performance or processing speed and visual attention. Therefore, the TMT-B finding should be considered exploratory and requires confirmation in larger, adequately powered randomized controlled trials.

## 1 Introduction

Population aging has intensified the demand for accessible, scalable, and non-pharmacological interventions that support cognitive health and functional independence in later life (United Nations Department of Economic and Social Affairs, Population Division, 2022; World Health Organization, 2020). Cognitive aging is characterized by declines across multiple domains, including memory, processing speed, attentional control, cognitive flexibility, and executive functioning, all of which play a critical role in maintaining autonomy, mobility, decision-making capacity, and engagement in everyday activities (Bherer, 2015; Livingston et al., 2024; Stuss and Craik, 2019). Among these domains, executive function is particularly important, as age-related impairments can compromise planning, inhibitory control, task switching, and adaptation to complex or dynamic environments (Bherer, 2015; Diamond and Ling, 2016). Consequently, validated instruments such as the Montreal Cognitive Assessment (MoCA) and the Trail Making Test (Parts A and B) are frequently employed in aging research to evaluate global cognitive performance, processing speed, visual attention, working memory, cognitive flexibility, and set-shifting abilities (Nasreddine et al., 2005; Tombaugh, 2004).

Physical exercise is widely recognized as one of the most effective lifestyle interventions for promoting cognitive and brain health in older adults (Bherer, 2013; Erickson et al., 2019; Stillman et al., 2020). Current international guidelines recommend regular engagement in aerobic, muscle-strengthening, balance, and multicomponent exercise because of their established benefits for physical function, functional independence, healthy aging, and disease prevention (Bull et al., 2020; World Health Organization, 2020). Accumulating evidence from meta-analyses indicates that exercise enhances cognitive performance in adults aged over 50 years, with consistent improvements reported in global cognition, memory, attention, and executive functions (Colcombe and Kramer, 2003; Northey et al., 2018; Falck et al., 2019). Moreover, higher levels of physical activity have been associated with a reduced risk of cognitive decline and dementia, highlighting exercise as a key modifiable factor for preserving cognitive health throughout aging (Iso-Markku et al., 2024; Livingston et al., 2024).

The cognitive effects of physical exercise can vary based on factors such as training type, intensity, duration, neuromotor complexity, and cognitive demands (Northey et al., 2018; Pesce, 2012; Stillman et al., 2020). Aerobic exercise is linked to better cardiorespiratory fitness, enhanced hippocampal plasticity, and improved memory functions in older adults (Erickson et al., 2011; ten Brinke et al., 2015). Resistance training may improve cognitive performance via various pathways, including neuromuscular, metabolic, vascular, neurotrophic, and executive-function mechanisms (Cassilhas et al., 2007; Coelho-Junior et al., 2022; Liu-Ambrose et al., 2010, 2012). However, the evidence is inconsistent, indicating that cognitive adaptations might be affected by factors like baseline cognitive status, exercise dose, intervention characteristics, and the cognitive outcomes measured (Falck et al., 2019; Stillman et al., 2020; Xu et al., 2023).

Multicomponent training (MCT) has emerged as a promising strategy to mitigate age-related cognitive decline because it combines strength, balance, coordination, gait, aerobic, and functional exercises within a single intervention (Forte et al., 2013; Suzuki et al., 2013; Eggenberger et al., 2015). By simultaneously engaging multiple physical and cognitive processes, MCT may place greater demands on attentional control, motor planning, task sequencing, postural stability, and adaptation to dynamic movement environments than single-modality exercise programs (Herold et al., 2018; Kim et al., 2020; Pesce, 2012). Evidence from randomized controlled trials and recent systematic reviews indicates that MCT can enhance global cognitive performance as well as specific cognitive domains in older adults, including individuals with mild cognitive impairment and cognitive frailty. However, the magnitude of these benefits appears to depend on factors such as intervention dosage, training composition, participant characteristics, and the cognitive assessment methods employed (Eggenberger et al., 2015; Liu et al., 2025; Luo et al., 2024).

Elastic-band training (EBT) is a practical, cost-effective, and widely accessible strength-training modality for older adults (Vásquez-Carrasco et al., 2025). Its portability, minimal equipment requirements, and adaptability to different functional capacities make it particularly suitable for community, home-, and low-resource settings (Hernández-Martinez et al., 2024a, 2024b; Li et al., 2024; Meng et al., 2025; Sampaio et al., 2020). Previous studies have demonstrated that EBT can improve muscle strength, balance, mobility, body composition, functional capacity, frailty-related outcomes, and performance in activities of daily living among older adults (Chen et al., 2016; Hernández-Martinez et al., 2024a, 2024b; Li et al., 2024; Saragih et al., 2022). However, its effects on cognitive function remain insufficiently understood. Although preliminary evidence suggests that EBT may enhance cognition and functional independence in institutionalized older adults, the available findings are limited and characterized by substantial heterogeneity (Sánchez-Lastra et al., 2022). Furthermore, compared with MCT, EBT may impose lower cognitive and motor demands, as MCT incorporates tasks requiring postural control, coordination, balance, gait, movement sequencing, and continuous cognitive–motor adaptation (Diamond and Ling, 2016; Herold et al., 2018; Pesce, 2012).

While there is growing evidence that exercise benefits cognitive health, earlier studies and systematic reviews have mostly assessed exercise, MCT, and EBT separately. As a result, there are few direct comparisons of MCT and EBT effects on cognitive outcomes in older women (Falck et al., 2019; Hernández-Martinez et al., 2024a, 2024b; Northey et al., 2018; Sánchez-Lastra et al., 2022; Xu et al., 2023). So far, there has been no randomized controlled trial that directly compares these interventions with executive function as the main outcome in older women living in the community. MCT includes a variety of activities such as aerobic, strength, balance, and functional exercises, offering a broad spectrum of cognitive-motor stimuli. In contrast, EBT primarily enhances muscular strength, neuromuscular function, and physical performance (Eggenberger et al., 2015; Forte et al., 2013; Liu-Ambrose et al., 2012; Sánchez-Lastra et al., 2022). This question is especially pertinent for older women, a group where cognitive decline, decreased physical function, and reduced muscle strength often occur together and influence one another (Hernández-Martinez et al., 2024a, 2024b; Liu-Ambrose et al., 2010; ten Brinke et al., 2015).

Therefore, this randomized controlled trial examined the effects of multicomponent training (MCT) and elastic-band training (EBT) on global cognition and executive function in older women. It was hypothesized that MCT would produce greater cognitive improvements than EBT, particularly in executive function domains involving working memory, cognitive flexibility, and set shifting.

## 2 Methods

### 2.1 Study design

This study was an outcome-assessor-blinded, parallel-group randomized controlled trial. Participants were randomly allocated to the MCT or EBT group in a 1:1 ratio using an online randomization program (Randomizer.org). The allocation sequence was generated by an investigator who was not involved in the outcome assessments, and the outcome assessor remained blinded to group allocation throughout the study.

The trial was reported in accordance with the CONSORT guidelines and registered at ClinicalTrials.gov (NCT0678002). Both interventions lasted 16 weeks and comprised 32 supervised 60-min sessions delivered twice weekly on nonconsecutive days. The EBT group trained on Mondays and Wednesdays, whereas the MCT group trained on Tuesdays and Thursdays.

Executive function, assessed using the Trail Making Test Part B (TMT-B), was designated as the primary outcome. Secondary outcomes included global cognitive performance, evaluated using the Montreal Cognitive Assessment (MoCA), and processing speed and visual attention, assessed using the Trail Making Test Part A (TMT-A). Analyses of the MoCA cognitive domains were considered exploratory.

Outcome assessments were conducted before and after the 16-week intervention under standardized laboratory conditions. All cognitive assessments were administered at both time points by the same trained assessor, who remained blinded to group allocation and followed standardized administration and scoring procedures. Assessments were conducted between 4:00 p.m. and 6:00 p.m. under identical conditions. Inter-rater reliability was not applicable because all assessments were administered by a single assessor, and no study-specific test–retest reliability coefficient was calculated. Procedural reproducibility was promoted through the use of standardized assessment conditions and validated Spanish-language versions of the cognitive instruments.

No musculoskeletal or cardiorespiratory adverse events were reported during the intervention, and no participant experienced pain during training sessions or immediately before the outcome assessments. A detailed overview of participant recruitment, allocation, follow-up, and analysis is provided in Figure 1.

**Figure 1:**
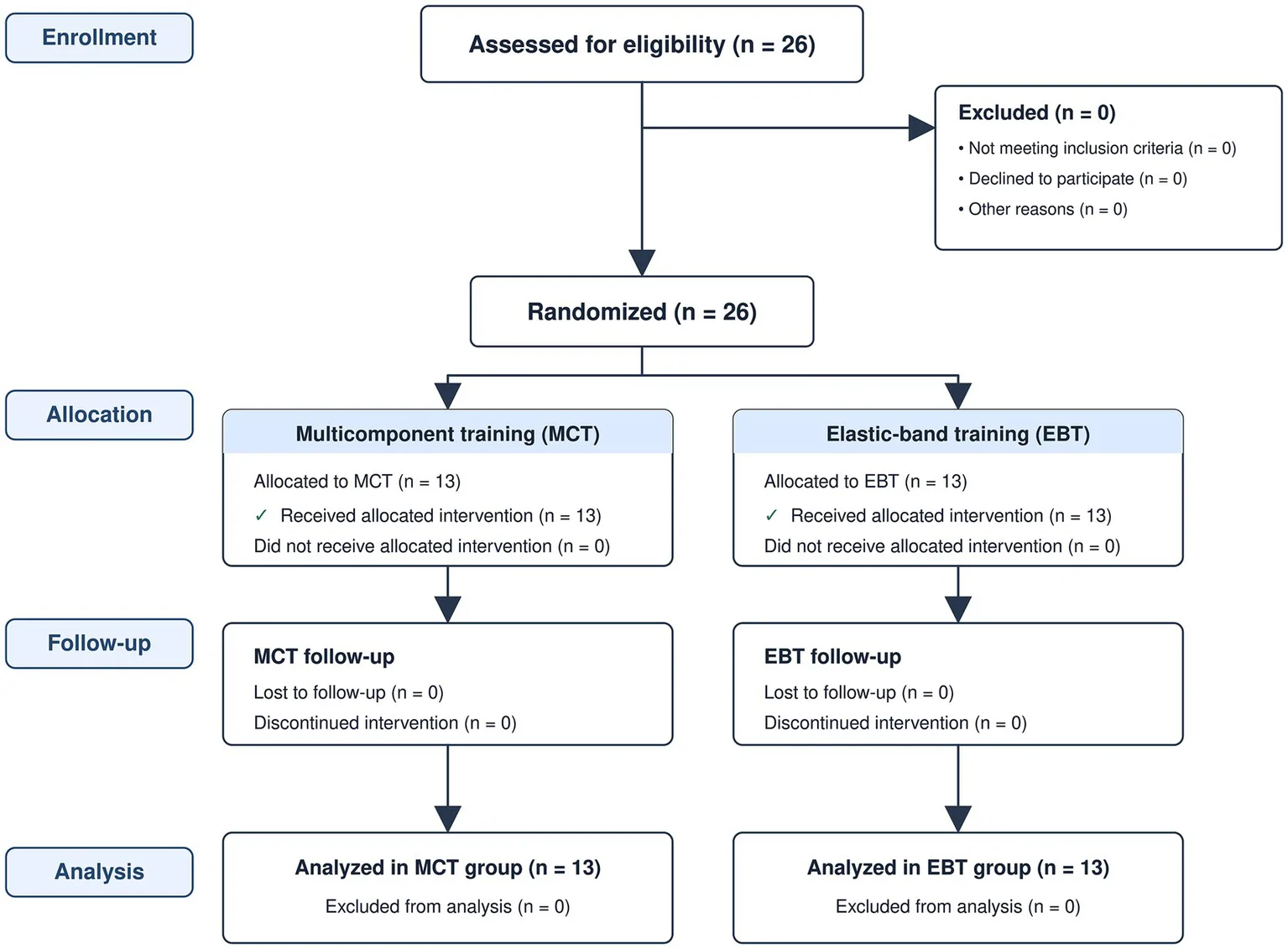
CONSORT flow diagram of participant enrollment, allocation, follow-up, and analysis.

### 2.2 Participants

Twenty-six older women were recruited and randomly assigned to either the EBT group (n = 13) or the MCT group (n = 13). Sample size adequacy was determined a priori using G*Power software (version 3.1.9.6; University of Kiel, Germany). Because the primary analysis involved comparing cognitive outcomes over time between groups, a repeated-measures ANOVA with a within–between interaction was selected as the most appropriate approximation. The calculation was informed by a previous randomized controlled trial investigating exercise-based interventions in older adults, which reported that 11 participants per group were sufficient to detect significant cognitive changes using a mixed-design approach (Guimarães et al., 2018). The analysis assumed two groups, two assessment time points, an alpha level of 0.05, statistical power of 80%, an effect size of f = 0.50, a correlation of 0.50 between repeated measures, and a sphericity correction factor (ε) of 1.00. Based on these parameters, the minimum required sample size was 22 participants (11 per group). Therefore, the final sample of 26 participants exceeded the estimated requirement and provided adequate power for the planned group × time analyses of cognitive outcomes.

The inclusion criteria were as follows: (i) women aged 65–75 years; (ii) ability to understand and follow contextually appropriate instructions through simple commands; (iii) functional independence, defined as a score of at least 43 points on the Preventive Medical Examination for Older People (in Spanish, EMPAM) from the Chilean Ministry of Health (Ministerio de Salud de Chile, n.d); and (iv) availability and commitment to attend at least 85% of the scheduled intervention sessions. The exclusion criteria were (i) the presence of any disability or health condition that limited safe participation in the intervention; (ii) musculoskeletal injury or ongoing physical rehabilitation that prevented participation in usual physical activity; and (iii) temporary or permanent medical contraindication to physical exercise.

All participants met the eligibility criteria, completed both baseline and post-intervention cognitive assessments, and were included in the final analyses. Written informed consent was obtained from each participant prior to enrollment, permitting the use of their data for research purposes. The study protocol was approved by the Scientific Ethics Committee of the Universidad Católica del Maule, Chile (Approval No. 29–2022), and all procedures were conducted in accordance with the principles outlined in the Declaration of Helsinki.

### 2.3 Anthropometric parameters and sociodemographic assessments

Height was measured to the nearest 0.1 cm using a wall-mounted stadiometer (Seca 220, SECA, Hamburg, Germany). Body mass was measured to the nearest 0.1 kg using a calibrated mechanical scale (Scale-Tronix, Chicago, IL, U*nited* S*tates*). Participants wore light clothing and no footwear, following standardized anthropometric protocols (Marfell-Jones et al., 2012). Sociodemographic data such as age, education level, and marital status were gathered at the beginning of the study. The baseline anthropometric and sociodemographic characteristics of the study participants are shown in Table 1.

**Table 1:** Baseline anthropometric parameters and sociodemographic assessments of older women.

Variable	Assessment	EBT (*n* = 13)	MCT (*n* = 13)
Age	Years	70.85 (±4.42)	69.92 (±4.17)
Anthropometric parameters	Body height (cm)	157 (±0.07)	158 (±0.06)
	Body mass (kg)	62.4 (±5.4)	65.5 (±4.69)
BMI	(kg/m^2^)	25.3 (±3.1)	26.2 (± 2.7)
Educational level	Primary	5	6
	Secondary	7	6
	Bachelor	1	1
	Postgraduate	0	0
Marital status	Married	12	10
	Separated	1	2
	Widowed	0	1
	Single	0	0

EBT: elastic band group; MCT: multicomponent group. Data are presented as the mean and standard deviation.

### 2.4 Montreal cognitive assessment

Brief cognitive screening instruments are commonly used to assess global cognitive functioning and identify mild cognitive impairment. The MoCA evaluates multiple cognitive domains, including visuospatial and executive function, naming, attention, language, abstraction, delayed recall, and orientation (Nasreddine et al., 2005). Scores range from 0 to 30, with higher values indicating better cognitive performance. Therefore, improvements in MoCA scores were interpreted as reflecting enhanced global cognitive functioning across the domains assessed. The inclusion of the MoCA in the present study was considered appropriate because it provides a comprehensive measure of overall cognitive status while complementing domain-specific assessments of processing speed and executive function. For Spanish-speaking populations, validated Spanish-language versions of the MoCA have demonstrated adequate psychometric properties for the detection of mild cognitive impairment and dementia among older adults in Latin America. Furthermore, validation and standardization studies conducted in the Chilean population support its use in both clinical and research settings (Gil et al., 2015; Delgado et al., 2019; Gaete et al., 2023).

### 2.5 Trail making test a

The TMT-A is a widely used neuropsychological measure of processing speed, visual scanning, sustained attention, visuomotor coordination, and motor response speed (Reitan, 1958; Tombaugh, 2004). Performance is typically quantified by completion time, with shorter times indicating greater psychomotor speed and attentional efficiency. Therefore, improvements in TMT-A performance were interpreted as reductions in the time required to sequentially identify and connect numerical stimuli while maintaining effective visual attention and motor execution. The inclusion of the TMT-A was particularly relevant because it assesses fundamental cognitive and motor processes that may contribute to performance on more demanding executive-function tasks, such as the TMT-B. Furthermore, the TMT-A has been extensively validated in Spanish-speaking populations and is supported by normative data for Latin American adults that account for key sociodemographic factors, including age, education, and sex, enhancing the interpretability of findings in both clinical and research settings (Arango-Lasprilla et al., 2015; Henao-Arboleda et al., 2010).

### 2.6 Trail making test B

Executive functioning was evaluated using the TMT-B, a task designed to assess higher-order cognitive processes involved in goal-directed behavior. Successful performance requires the integration of several executive abilities, including the maintenance and updating of information, suppression of inappropriate responses, set shifting, and strategic planning during cognitively demanding activities (Reitan, 1958). Because outcomes are based on the time needed to complete the task, lower scores represent more efficient executive processing. Therefore, decreases in completion time were interpreted as reflecting enhanced capacity to coordinate multiple cognitive operations, switch between mental sets, adhere to task requirements, and regulate responses efficiently. The Spanish adaptation of the TMT-B has demonstrated adequate psychometric properties, with reported reliability coefficients supporting its use in both clinical and research contexts. Moreover, normative reference values derived from Latin American populations facilitate the interpretation of performance among Spanish-speaking adults (Henao-Arboleda et al., 2010; Arango-Lasprilla et al., 2015).

### 2.7 Intervention

Participants completed a 16-week exercise program comprising 32 supervised sessions performed twice weekly on nonconsecutive days at the functional training facilities of the sports center at Universidad Autónoma de Chile. The interventions were delivered by postgraduate gerontology trainees with previous experience in exercise programs for older adults, under the supervision of the study investigator. Before each session, resting heart rate and systolic and diastolic blood pressure were measured using an automated monitor (HEM-7120; OMRON, Japan) as part of the pre-exercise safety screening.

Each session lasted approximately 60 min and followed the same standardized structure in both groups. Sessions began with a 10-min warm-up comprising joint mobility and low-intensity aerobic activities, such as treadmill walking, stationary cycling, and instructor-led activation exercises. The 40-min main phase was organized into two alternating full-body routines performed on the two weekly training days. Each routine comprised six to eight stations arranged to alternate upper-limb, lower-limb, balance or agility, and cardiorespiratory exercises. Strength exercises were prescribed by repetitions, whereas balance, agility, and cardiorespiratory tasks were performed for 30–60 s, depending on the exercise. Sessions concluded with a 10-min cool-down comprising dynamic and static stretching.

One complete circuit consisted of performing each prescribed station once. During weeks 1–4, participants completed three circuits, with 10 repetitions at each strength station. During weeks 5–8, the volume increased to four circuits of 10 repetitions. During weeks 9–12, participants performed four circuits of 12 repetitions, and this volume was maintained during weeks 13–16. Rest intervals of 120 s were provided between circuits during weeks 1–12 and were reduced to 90 s during weeks 13–16. Resistance exercises were performed at a controlled tempo of approximately 2 s during the concentric phase and 4 s during the eccentric phase.

Exercise intensity was monitored using the OMNI-Resistance Exercise Scale (Lagally and Robertson, 2006; Petro et al., 2025), with a target perceived exertion of 5–8. Initial resistance was individualized to maintain participants within this range. When a participant completed all prescribed repetitions with correct technique and reported an OMNI rating below 5, resistance was increased using the smallest available weight increment in MCT or the next band-resistance level in EBT. Band tension could also be increased by shortening the working length of the band. Resistance was maintained or reduced when perceived exertion exceeded 8 or movement quality deteriorated. The session structure and progression of the intervention are summarized in Figure 2.

**Figure 2:**
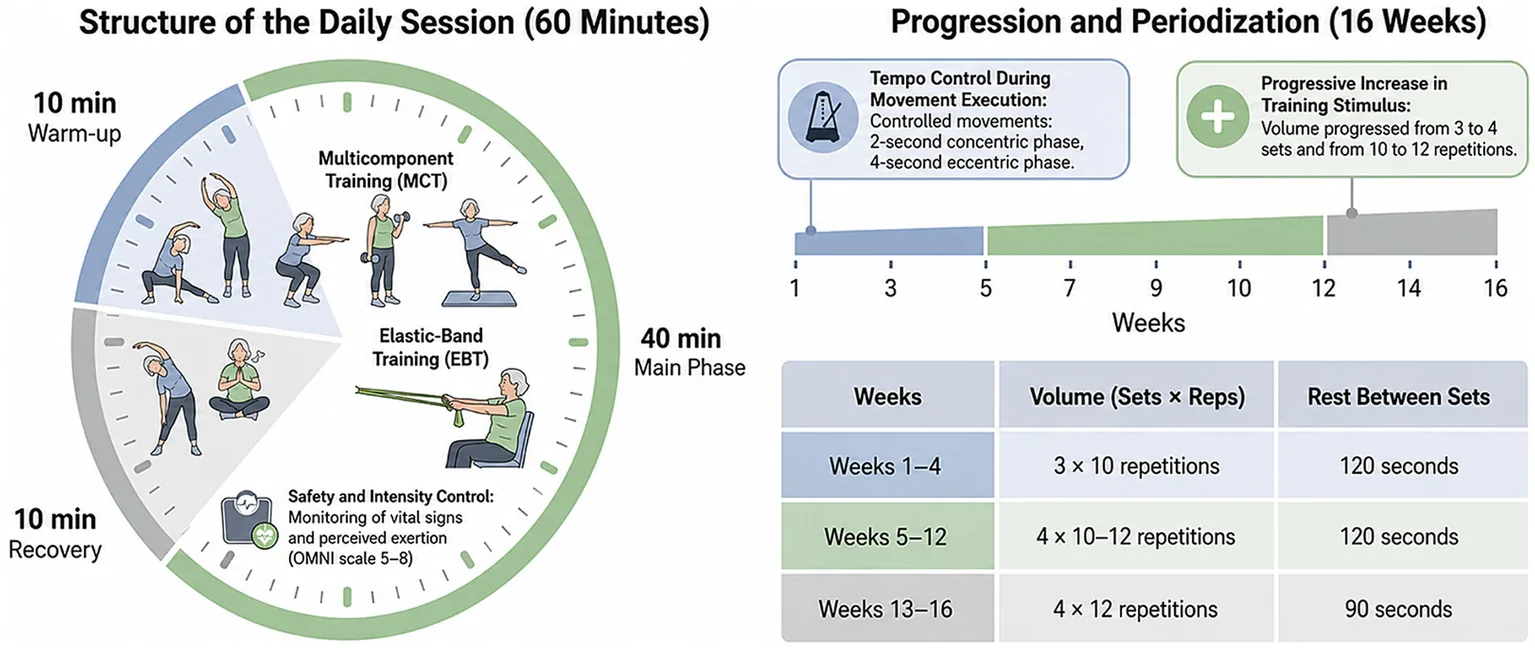
Daily session structure and progression of the 16-week intervention program.

### 2.8 Multicomponent training program

The MCT main phase combined strength, balance, agility, and cardiorespiratory exercises. Strength exercises included squats, sit-to-stand exercises, step-ups, heel raises, biceps curls, triceps extensions, shoulder abductions and presses, and rows. Balance exercises comprised semi-tandem and tandem stances, single-leg stance, heel-to-toe walking, multidirectional weight shifting and reaching, and stepping over obstacles. Agility exercises included slalom walking between cones, lateral stepping, changes of direction, forward and backward walking, and responses to verbal cues. Cardiorespiratory activities consisted of marching in place and low-impact aerobics circuits. Exercises were performed using body weight and external equipment, including dumbbells, kettlebells, poles, and medicine balls. Exercises were distributed between the two alternating routines and arranged to vary the muscular and motor demands across consecutive stations.

### 2.9 Elastic band training program

The EBT program was based on previous evidence supporting its safety, feasibility, and effectiveness in older adults (Choi et al., 2020; Hernández-Martinez et al., 2024a, 2024b; Valdés-Badilla et al., 2023). Training was performed using the Ultimate Elastic Resistance Band System Elite. The bands were color-coded (yellow, red, green, and black), with each color representing a different resistance level.

The program included 12 exercises divided between two alternating six-exercise routines, allowing all exercises to be completed over each training week. Upper-limb exercises included lat pulldown, scapular retraction, shoulder abduction, biceps curl, triceps extension, and upright row. Lower-limb exercises included leg press, ankle eversion, ankle dorsiflexion, knee extension, knee flexion, and hip flexion. Participants began with the lowest appropriate resistance level and progressed according to perceived exertion, movement quality, and their ability to complete the prescribed repetitions.

### 2.10 Statistical analysis

Cognitive outcomes were analyzed using the R statistical environment (version 4.5.1; R Foundation for Statistical Computing, Vienna, Austria) through the RStudio interface (RStudio Team, Boston, MA, U**nited** S**tates**). Changes occurring throughout the intervention period were investigated separately for global cognition, cognitive-domain scores derived from the MoCA, and executive-function measures obtained from the TMT.

Repeated observations collected from the same participants were handled using multilevel modeling procedures that accounted for within-subject dependence. Intervention condition (EBT or MCT), assessment occasion (baseline and post-intervention), and their combined effect were incorporated into each model. Individual participants were treated as clustering units through subject-specific intercepts, whereas age and educational attainment were entered to adjust for potential confounding influences.

The main confirmatory analysis focused on TMT-B performance because this outcome was considered the most sensitive indicator of executive-function adaptations and was directly linked to the study hypothesis. Specifically, it was expected that participants exposed to MCT would demonstrate greater improvements in cognitive flexibility and task-switching abilities than those assigned to EBT. Global MoCA performance and TMT-A scores were evaluated as secondary endpoints, while analyses involving MoCA-derived domains were considered exploratory.

Statistical evidence for model effects was obtained using Type III tests with Satterthwaite-adjusted degrees of freedom. Whenever intervention-related changes differed across assessment occasions, follow-up contrasts based on model-derived marginal estimates were used to characterize the direction and magnitude of these differences. Comparisons were conducted both within intervention groups and between interventions at each evaluation point.

To limit inflation of false-positive findings arising from multiple testing, false discovery rate procedures were applied to exploratory domain analyses and follow-up comparisons. In accordance with the predefined analytical hierarchy, no multiplicity adjustment was imposed on the primary TMT-B interaction effect. Nevertheless, a supplementary analysis incorporating false discovery rate correction across all interaction terms was performed to examine the stability of the results.

Effect magnitude was summarized using partial eta squared (η^2^p). Values near 0.01, 0.06, and 0.14 were interpreted as small, moderate, and large effects, respectively (Richardson, 2011). Prior to model estimation, completion times for TMT-A and TMT-B were logarithmically transformed because their distributions deviated from assumptions of normality and variance homogeneity. Statistical evidence was considered sufficient when two-sided probability values were below 0.05.

## 3 Results

Twenty-six older women completed the intervention and were included in the final analyses, with 13 participants assigned to the EBT group and 13 to the MCT group. The mean age was 70.85 ± 4.42 years in the EBT group and 69.92 ± 4.17 years in the MCT group. Baseline global cognitive function was similar between groups, with MoCA scores of 20.8 ± 6.5 and 20.5 ± 3.7 points in the EBT and MCT groups, respectively. Baseline anthropometric and sociodemographic characteristics are detailed in Table 1. The results of the linear mixed-effects models, along with the estimated marginal means for all cognitive outcomes, are presented in Table 2.

**Table 2:** Results of linear mixed-effects models, observed descriptive statistics, and estimated marginal means.

Variable	F (group × time)	*p*	MCT pre observed/EMM (95% CI)	EBT pre observed/EMM (95% CI)	MCT post observed/EMM (95% CI)	EBT post observed/EMM (95% CI)	Δ MCT (EMM)	Δ EBT (EMM)
MoCA total	1.18	0.289	20.54 ± 3.71/14.48 (10.57–18.39)	20.79 ± 6.52/14.51 (11.44–17.58)	22.62 ± 4.27/16.34 (12.38–20.31)	21.23 ± 6.81/15.28 (12.02–18.53)	1.86	0.77
Visuospatial	0.62	0.439	0.85 ± 0.69/0.68 (0.41–0.95)	1.21 ± 0.79/0.61 (0.36–0.86)	1.08 ± 0.64/0.68 (0.43–0.94)	1.00 ± 0.91/0.73 (0.43–1.02)	0.00	0.12
Clock	0.94	0.341	2.00 ± 1.08/1.11 (0.71–1.50)	2.05 ± 0.71/1.13 (0.81–1.45)	2.00 ± 0.91/1.08 (0.71–1.45)	1.92 ± 1.19/1.18 (0.76–1.60)	−0.03	0.05
Naming	0.71	0.408	2.69 ± 0.63/2.13 (1.89–2.37)	2.63 ± 0.60/2.25 (2.02–2.47)	2.46 ± 0.66/1.91 (1.66–2.16)	2.31 ± 0.85/2.25 (1.96–2.54)	−0.22	0.00
Attention	0.84	0.368	2.69 ± 1.65/1.54 (0.92–2.15)	3.05 ± 1.96/1.83 (1.22–2.44)	3.77 ± 1.92/2.72 (2.11–3.33)	4.08 ± 1.89/2.98 (2.41–3.56)	1.18	1.15
Language	1.92	0.179	1.15 ± 1.21/0.55 (0.07–1.02)	1.32 ± 1.16/0.58 (0.13–1.04)	1.92 ± 0.95/1.27 (0.88–1.65)	1.38 ± 0.96/0.74 (0.36–1.12)	0.72	0.16
Abstraction	2.64	0.117	1.08 ± 0.76/0.60 (0.31–0.89)	0.74 ± 0.87/0.35 (0.02–0.67)	1.23 ± 0.83/0.73 (0.42–1.04)	1.23 ± 0.83/0.98 (0.67–1.28)	0.14	0.63
Delayed recall	0.53	0.474	3.46 ± 1.20/2.82 (2.37–3.27)	3.58 ± 1.46/2.64 (2.21–3.07)	3.92 ± 0.95/2.40 (2.02–2.78)	3.54 ± 1.51/2.64 (2.18–3.10)	−0.42	0.00
Orientation	5.86	0.024*	5.85 ± 0.38/4.92 (4.74–5.11)	5.47 ± 1.02/4.79 (4.60–4.98)	5.92 ± 0.28/4.92 (4.73–5.11)	5.38 ± 0.96/4.51 (4.32–4.70)	0.00	−0.28
TMT-A log(s)	1.61	0.214	4.99 ± 0.42/4.92 (4.69–5.16)	4.67 ± 0.40/5.02 (4.78–5.26)	4.42 ± 0.29/4.76 (4.52–5.00)	4.80 ± 0.56/5.02 (4.79–5.26)	−0.16	0.00
TMT-B log(s)	6.58	0.019*	5.36 ± 0.44/5.22 (4.99–5.45)	5.25 ± 0.55/5.20 (4.97–5.42)	5.21 ± 0.30/5.05 (4.84–5.27)	5.25 ± 0.67/5.40 (5.19–5.62)	−0.17	0.20

MoCA: Montreal Cognitive Assessment; TMT-A: Trail Making Test Part A; TMT-B: Trail Making Test Part B. Values are presented as observed mean ± standard deviation (SD)/estimated marginal mean (EMM) with 95% confidence interval (CI). Δ represents the change from pre to postintervention. F and *p* values correspond to the model-level group × time interaction. TMT-B was specified as the primary executive-function outcome; MoCA total score and TMT-A were secondary outcomes; and MoCA-derived domains were exploratory outcomes. False discovery rate correction was applied to post hoc pairwise comparisons and exploratory MoCA-domain analyses. For TMT outcomes, lower values indicate better performance.* represents *p* < 0.05.

### 3.1 Montreal cognitive assessment

Analysis of the MoCA outcomes revealed no evidence of between-group differences for any cognitive measure (all *p* > 0.05). Furthermore, adjustment for multiple testing using the false discovery rate procedure showed that changes over time were comparable between groups across the overall MoCA score and nearly all cognitive subdomains.

The global cognitive score did not exhibit differential changes throughout the intervention period [*F*(1,24) = 1.18, *p* = 0.289, η^2^p = 0.047]. Likewise, trajectories for visuospatial performance (*F* = 0.62, *p* = 0.439, η^2^p = 0.025), clock-drawing ability (*F* = 0.94, *p* = 0.341, η^2^p = 0.038), naming (*F* = 0.71, *p* = 0.408, η^2^p = 0.029), attention (*F* = 0.84, *p* = 0.368, η^2^p = 0.034), language (*F* = 1.92, *p* = 0.179, η^2^p = 0.074), abstraction (*F* = 2.64, *p* = 0.117, η^2^p = 0.099), and delayed recall (*F* = 0.53, *p* = 0.474, η^2^p = 0.021) remained statistically comparable between the intervention conditions. Magnitudes of the observed effects were generally small, although language and abstraction approached the moderate range.

Of all domains examined, orientation produced the largest interaction estimate (*F* = 5.86, *p* = 0.024, η^2^p = 0.196). Nevertheless, this apparent difference was no longer supported after controlling for multiple comparisons.

### 3.2 Trail making test a

No significant main effects of group or time × group interactions were found for TMT-A performance [*F*(1,24) = 1.61, *p* = 0.214, η^2^p = 0.063]. Likewise, no significant between-group differences were detected at either baseline or post-intervention after false discovery rate (FDR) correction (all pFDR > 0.05). Within-group analyses also showed no significant pre–post changes in either intervention group (all pFDR > 0.05).

### 3.3 Trail making test B

Linear mixed-model analysis demonstrated a significant group × time interaction for TMT-B performance, the primary measure of executive function [*F*(1, 19.38) = 6.58, *p* = 0.019, η^2^p = 0.253], corresponding to a large effect size. Estimated marginal means suggested distinct temporal patterns between groups, with the MCT group exhibiting faster TMT-B completion times over the intervention period, whereas performance declined in the EBT group.

Despite this interaction, *post hoc* comparisons based on estimated marginal means revealed no significant between-group differences at either baseline or postintervention after FDR adjustment (all pFDR > 0.05). Similarly, within-group pre–post changes were not significant following correction for multiple testing. Furthermore, in a conservative sensitivity analysis applying FDR correction across all model-level group × time interactions, the TMT-B interaction no longer reached statistical significance.

Taken together, these findings support the presence of a significant model-level interaction and indicate differential trajectories of executive-function performance between groups. However, they do not provide sufficient evidence to conclude a superior postintervention effect of MCT compared with EBT.

## 4 Discussion

The present RCT found no significant intervention effects on global cognition, as measured by the MoCA, or on TMT-A performance. However, a significant group × time interaction was observed at the model level for TMT-B, indicating a potentially more favorable trajectory of executive function following MCT. Estimated marginal means showed a decrease in TMT-B completion time in the MCT group, whereas performance declined in the EBT group over the intervention period. Adjusted *post hoc* comparisons did not reach statistical significance, and the interaction effect was no longer significant after applying a conservative FDR correction across all model-level interactions. Consequently, these findings should be interpreted as evidence of differing executive-function trajectories between interventions rather than conclusive proof of MCT superiority. Although no significant improvements were observed in MoCA or TMT-A performance, the observed stability in global cognition, processing speed, and visual attention over 16 weeks may be descriptively relevant in older women, given their vulnerability to age-related cognitive decline (Stuss and Craik, 2019). However, this stability should not be interpreted as a protective intervention effect because no non-exercising control group was included and practice effects arising from repeated assessments cannot be excluded.

The cognitive trends observed in this study are consistent with the notion that exercise programs combining multiple physical capacities may provide greater cognitive stimulation than single-modality approaches. Interventions that simultaneously challenge balance, coordination, aerobic fitness, and functional performance have been associated with maintenance or improvement of specific cognitive abilities in older adults, particularly among individuals with increased susceptibility to cognitive decline and when training tasks require active cognitive engagement (Eggenberger et al., 2015; Kim et al., 2020; Luo et al., 2024; Wang et al., 2024). Within this context, the more favourable evolution of TMT-B performance may be explained by the higher cognitive demands inherent to MCT, which requires the continuous integration of motor and executive processes, rather than by physical exercise exposure per se (Voelcker-Rehage and Niemann, 2013; Wollesen and Voelcker-Rehage, 2014). Conversely, the absence of significant improvements in MoCA and TMT-A scores may indicate that these measures are less sensitive to subtle, short-term cognitive adaptations associated with exercise interventions. Because TMT-B places greater demands on cognitive flexibility, task switching, and executive control, it may be more capable of detecting changes elicited by training activities that involve ongoing motor adaptation and complex movement sequencing (Herold et al., 2018).

Evidence from EBT interventions provides an important point of comparison. Previous studies have demonstrated that EBT improves muscle strength, balance, mobility, functional performance, and activities of daily living in older adults, supporting its role as a practical and cost-effective exercise modality for aging populations (Chen et al., 2016; Saragih et al., 2022; Li et al., 2024; Hernández-Martinez et al., 2024a, 2024b). However, its effects on cognitive outcomes remain less well established. Although EBT may contribute to improvements in cognition and functional independence among institutionalized older adults, the cognitive demands of this modality may be lower than those of MCT, particularly when training sessions incorporate fewer concurrent challenges related to balance, coordination, movement sequencing, and task switching (Pesce, 2012; Sánchez-Lastra et al., 2022). Alternatively, the between-group differences observed in the present study may reflect an insufficient level of cognitive-motor stimulation in the EBT program to maintain executive-function performance over time, rather than a uniquely enhancing effect of MCT.

From a neurocognitive perspective, the more favorable TMT-B trajectory after MCT may be explained by the greater executive demands embedded in this modality (Herold et al., 2018; Raichlen and Alexander, 2017). MCTs require the continuous integration of balance control, coordination, gait adjustments, postural regulation, sequencing, attentional allocation, and adaptation to changing task constraints (Seidler et al., 2010; Wollesen and Voelcker-Rehage, 2014). These demands may preferentially engage the prefrontal and frontoparietal networks involved in cognitive flexibility, set-shifting, inhibition, and goal-directed control (Diamond and Ling, 2016; Miller and Cohen, 2001). In contrast, EBT primarily activates neuromuscular and strength-related mechanisms, with comparatively lower demands on simultaneous cognitive–motor coordination (Fragala et al., 2019; Herold et al., 2019). This may explain why the observed pattern was domain specific, with stable global cognition and processing speed but a differential trajectory in executive function performance (Chen et al., 2020; Northey et al., 2018).

At the neurobiological level, exercise may enhance cognitive function through multiple interacting mechanisms, including improved cerebrovascular regulation, increased neurotrophic activity, enhanced synaptic plasticity, metabolic adaptations, and structural brain remodeling (Erickson et al., 2019; Voss et al., 2013). Different exercise modalities may influence these pathways through distinct mechanisms. Aerobic and multicomponent training appear to facilitate hippocampal and cortical plasticity, whereas resistance-based exercise may exert its effects through neuromuscular activation, vascular adaptations, and engagement of executive-control networks (Cassilhas et al., 2007; Erickson et al., 2011; ten Brinke et al., 2015; Coelho-Junior et al., 2022).

Within this framework, the present findings may indicate a domain-specific cognitive response to exercise. Global cognitive performance remained largely unchanged, and processing speed showed minimal variation; however, executive function followed a more favorable trajectory in the intervention characterized by greater cognitive–motor complexity. These results highlight the importance of considering not only exercise dosage but also qualitative training attributes, such as coordinative demands, movement variability, task sequencing, postural control, and cognitive engagement, when designing interventions aimed at preserving cognitive health in older women (Pesce, 2012; Stillman et al., 2020).

### 4.1 Strengths and limitations

This study has several notable strengths. First, its randomized controlled design enabled a direct comparison of two accessible and cost-effective exercise interventions in older women, a population particularly vulnerable to the combined effects of cognitive decline, reduced physical function, and loss of muscle strength. Second, the assessment of both global cognition and executive function provided a more comprehensive evaluation of cognitive outcomes, revealing domain-specific adaptations that may not be detected through global screening measures alone. Third, the use of linear mixed-effects models allowed robust estimation of intervention effects over time by accounting for within-subject correlations and evaluating group, time, and interaction effects. Finally, the comparison of multicomponent and elastic-band training has clear clinical relevance, as both approaches are practical, scalable, and readily implementable in community, rehabilitation, and resource-constrained settings.

Several limitations should be considered when interpreting these findings. Although the final sample exceeded the minimum estimated for the prespecified group × time analysis, the total sample of 26 participants provided limited power for *post hoc* comparisons, particularly after correction for multiple testing. Accordingly, adjusted comparisons and effect-size estimates should be interpreted with caution. The twice-weekly frequency also represented a relatively modest exercise dose, which may have limited detectable changes in MoCA and TMT-A performance. Furthermore, the inclusion of only older women limits generalizability to men and more diverse populations. The absence of a non-exercising control group prevents distinguishing intervention-related changes from practice effects arising from repeated cognitive assessments; therefore, the observed maintenance or changes cannot be attributed exclusively to either intervention. The lack of follow-up assessments also prevents conclusions regarding the durability of the findings. Finally, because cognition was evaluated solely through behavioral measures, the mechanisms underlying the observed TMT-B trajectory remain speculative.

### 4.2 Practical applications

The present findings suggest that MCT may be an effective approach for enhancing executive function in older women, particularly cognitive flexibility and set-shifting abilities. From a practical standpoint, exercise prescription for older adults should consider not only traditional training variables such as intensity, duration, and frequency but also the cognitive–motor demands of the intervention (Vásquez-Carrasco et al., 2026). Activities that require balance, coordination, postural adjustments, gait adaptation, movement sequencing, and responses to changing task demands may provide greater stimulation of executive processes.

EBT remains a practical and accessible exercise modality due to its low cost, portability, ease of individualization, and suitability for home-, community-, and resource-limited settings. However, when cognitive enhancement is a primary therapeutic objective, EBT may benefit from the incorporation of cognitively demanding elements, such as dual-task exercises, variable movement patterns, balance challenges, and decision-making tasks. These findings therefore support MCT as a potentially more cognitively stimulating intervention while reaffirming the value of EBT for maintaining muscular strength, mobility, and overall functional capacity.

For clinicians and exercise professionals, the TMT-B appears to be a particularly sensitive measure for detecting exercise-induced changes in executive function among older adults. In contrast, global cognitive screening instruments such as the MoCA may be less responsive to short-term cognitive adaptations in cognitively healthy individuals. Accordingly, future intervention studies and clinical programs should incorporate executive function assessments when targeting domains such as cognitive flexibility, attentional control, working memory, and set shifting.

### 4.3 Epidemiological applications

From an epidemiological perspective, these findings are noteworthy given the rapid growth of the aging population and the increasing demand for scalable interventions that support cognitive and functional health in later life. Although the present trial was not designed to assess dementia prevention or population-level outcomes, the observed benefits reinforce the public health rationale for incorporating structured exercise into healthy aging strategies. Physical activity is recognized as a modifiable factor associated with a reduced risk of cognitive decline and dementia, while exercise-based interventions are widely recommended because of their positive effects on physical function, autonomy, and overall healthy aging (Bull et al., 2020; Livingston et al., 2024; World Health Organization, 2020).

The comparison of MCT and EBT also carries potential public health relevance, as both approaches are scalable and feasible for implementation in community-based settings with minimal infrastructure demands. MCT may be particularly valuable for interventions targeting cognitive–motor health, whereas EBT offers an accessible alternative for individuals with limited resources, mobility constraints, or restricted access to supervised exercise programs. Consequently, the inclusion of cognitively enriched multicomponent exercise and accessible strength-focused training within community health initiatives may help preserve functional independence and mitigate the growing burden of age-related cognitive and physical decline.

Nevertheless, these implications should be interpreted with caution. The current findings should be considered exploratory and require confirmation through larger randomized trials with longer follow-up periods and more heterogeneous populations. Future studies should incorporate outcomes related to activities of daily living, incident cognitive impairment, healthcare utilization, disability, and long-term functional trajectories. In addition, epidemiological and implementation research is needed to determine whether scalable exercise interventions can generate sustained cognitive and functional benefits at the population level, particularly among older women and individuals at elevated risk of cognitive or functional deterioration.

## 5 Conclusion

In this study, neither intervention produced substantial changes in overall cognitive status or processing speed, as performance remained largely unchanged across the assessment period. A different pattern emerged for executive function. Specifically, the trajectory of TMT-B scores suggested a potential advantage for participants assigned to MCT, particularly in cognitive processes related to mental flexibility and task switching. However, this pattern should be interpreted with caution. Although the group-by-time interaction reached statistical significance in the primary model, subsequent adjusted comparisons failed to identify significant between-group differences, and the effect was no longer evident after controlling for multiple testing using a false discovery rate procedure.

Taken together, these findings provide only tentative support for differential effects of MCT and EBT on executive functioning. Rather than demonstrating a clear superiority of one training modality, the results indicate the possibility of distinct cognitive response patterns that warrant further investigation. The absence of comparable effects in global cognitive measures further suggests that exercise-related adaptations may not occur uniformly across cognitive domains and could be more readily detected in tasks that place greater demands on executive control.

Future research should include larger samples, extended intervention and follow-up periods, and a broader range of cognitive, functional, and biological outcomes. Such studies would help determine the robustness of the observed trends and establish whether they translate into clinically meaningful and population-level benefits.

## Data Availability

The raw data supporting the conclusions of this article will be made available by the authors, without undue reservation.

## References

[ref1] Arango-Lasprilla J. C. Rivera D. Aguayo A. Rodríguez W. Garza M. T. Saracho C. P. . (2015). Trail making test: normative data for the Latin American Spanish speaking adult population. NeuroRehabilitation 37, 639–661. doi: 10.3233/NRE-151284, 26639932

[ref2] Bherer L. (2013). A review of the effects of physical activity and exercise on cognitive and brain functions in older adults. J. Aging Res. 2013:657508. doi: 10.1155/2013/657508 PMC3786463 24102028

[ref3] Bherer L. (2015). Cognitive plasticity in older adults: effects of cognitive training and physical exercise. Ann. N. Y. Acad. Sci. 1337, 1–6. doi: 10.1111/nyas.12682, 25773610

[ref4] Bull F. C. Al-Ansari S. S. Biddle S. Borodulin K. Buman M. P. Cardon G. . (2020). World Health Organization 2020 guidelines on physical activity and sedentary behaviour. Br. J. Sports Med. 54, 1451–1462. doi: 10.1136/bjsports-2020-102955, 33239350 PMC7719906

[ref5] Cassilhas R. C. Viana V. A. R. Grassmann V. Santos R. T. Santos R. F. Tufik S. . (2007). The impact of resistance exercise on the cognitive function of the elderly. Med. Sci. Sports Exerc. 39, 1401–1407. doi: 10.1249/mss.0b013e318060111f, 17762374

[ref6] Chen F. T. Etnier J. L. Chan K. H. Chiu P. K. Hung T. M. Chang Y. K. (2020). Effects of exercise training interventions on executive function in older adults: a systematic review and meta-analysis. Sports Med. 50, 1451–1467. doi: 10.1007/s40279-020-01292-x, 32447717 PMC7376513

[ref7] Chen K. M. Li C. H. Huang H. T. Cheng Y. Y. (2016). Feasible modalities and long-term effects of elastic band exercises in nursing home older adults in wheelchairs: a cluster randomized controlled trial. Int. J. Nurs. Stud. 55, 4–14. doi: 10.1016/j.ijnurstu.2015.11.004, 26655368

[ref8] Choi H. M. Hurr C. Kim S. (2020). Effects of elastic band exercise on functional fitness and blood pressure response in the healthy elderly. Int. J. Environ. Res. Public Health 17:7144. doi: 10.3390/ijerph17197144, 33003575 PMC7579118

[ref9] Coelho-Junior H. J. Marzetti E. Calvani R. Picca A. Arai H. Uchida M. C. (2022). Resistance training improves cognitive function in older adults with different cognitive status: a systematic review and meta-analysis. Aging Ment. Health 26, 213–224. doi: 10.1080/13607863.2020.1857691, 33325273

[ref10] Colcombe S. Kramer A. F. (2003). Fitness effects on the cognitive function of older adults: a meta-analytic study. Psychol. Sci. 14, 125–130. doi: 10.1111/1467-9280.t01-1-01430, 12661673

[ref11] Delgado C. Araneda A. Behrens M. I. (2019). Validation of the Spanish-language version of the Montreal cognitive assessment test in adults older than 60 years. Neurologia 34, 376–385. doi: 10.1016/j.nrl.2017.01.013, 28364958

[ref12] Diamond A. Ling D. S. (2016). Conclusions about interventions, programs, and approaches for improving executive functions that appear justified and those that, despite much hype, do not. Dev. Cogn. Neurosci. 18, 34–48. doi: 10.1016/j.dcn.2015.11.005, 26749076 PMC5108631

[ref13] Eggenberger P. Schumacher V. Angst M. Theill N. de Bruin E. D. (2015). Does multicomponent physical exercise with simultaneous cognitive training boost cognitive performance in older adults? A 6-month randomized controlled trial with a 1-year follow-up. Clin. Interv. Aging 10, 1335–1349. doi: 10.2147/CIA.S87732, 26316729 PMC4544626

[ref14] Erickson K. I. Hillman C. Stillman C. M. Ballard R. M. Bloodgood B. Conroy D. E. . (2019). Physical activity, cognition, and brain outcomes: a review of the 2018 physical activity guidelines. Med. Sci. Sports Exerc. 51, 1242–1251. doi: 10.1249/MSS.0000000000001936, 31095081 PMC6527141

[ref15] Erickson K. I. Voss M. W. Prakash R. S. Basak C. Szabo A. Chaddock L. . (2011). Exercise training increases size of hippocampus and improves memory. Proc. Natl. Acad. Sci. USA 108, 3017–3022. doi: 10.1073/pnas.1015950108, 21282661 PMC3041121

[ref16] Falck R. S. Davis J. C. Best J. R. Crockett R. A. Liu-Ambrose T. (2019). Impact of exercise training on physical and cognitive function among older adults: a systematic review and meta-analysis. Neurobiol. Aging 79, 119–130. doi: 10.1016/j.neurobiolaging.2019.03.007, 31051329

[ref17] Forte R. Boreham C. A. G. Leite J. C. De Vito G. Brennan L. Gibney E. R. . (2013). Enhancing cognitive functioning in the elderly: multicomponent vs resistance training. Clin. Interv. Aging 8, 19–27. doi: 10.2147/CIA.S36514 PMC3546758 23341738

[ref18] Fragala M. S. Cadore E. L. Dorgo S. Izquierdo M. Kraemer W. J. Peterson M. D. . (2019). Resistance training for older adults: position statement from the National Strength and conditioning association. J. Strength Cond. Res. 33, 2019–2052. doi: 10.1519/JSC.0000000000003230, 31343601

[ref19] Gaete M. Jorquera S. Bello-Lepe S. Mendoza Y. M. Véliz M. Alonso-Sánchez M. F. . (2023). Resultados estandarizados del Montreal Cognitive Assessment (MoCA) para cribado neurocognitivo en población chilena. Neurologia 38, 246–255. doi: 10.1016/j.nrl.2020.08.017, 33160725

[ref20] Gil L. Ruiz de Sánchez C. Gil F. Romero S. J. Pretelt Burgos F. (2015). Validation of the Montreal cognitive assessment (MoCA) in Spanish as a screening tool for mild cognitive impairment and mild dementia in patients over 65 years old in Bogotá, Colombia. Int. J. Geriatr. Psychiatry 30, 655–662. doi: 10.1002/gps.4199, 25320026

[ref21] Guimarães A. V. Barbosa A. R. Meneghini V. (2018). Active videogame-based physical activity vs. aerobic exercise and cognitive performance in older adults: a randomized controlled trial. J. Phys. Educ. Sport. 18, 203–209. doi: 10.7752/jpes.2018.01026

[ref22] Henao-Arboleda E. Muñoz C. Aguirre-Acevedo D. C. Lara E. Pineda D. A. Lopera F. (2010). Datos normativos de pruebas neuropsicológicas en adultos mayores en una población colombiana. Rev. Chil. Neuropsicol. 5, 213–225.

[ref23] Hernández-Martinez J. Cid-Calfucura I. Chiguay C. Weinberger M. Delgado-Floody P. Muñoz-Vásquez C. . (2024a). Effects of elastic band training on body composition and physical performance in older people: a systematic review with meta-analysis. Exp. Gerontol. 196:112553. doi: 10.1016/j.exger.2024.112553, 39197674

[ref24] Hernández-Martinez J. Guzmán-Muñoz E. Cid-Calfucura I. Villalobos-Fuentes F. Diaz-Saldaña D. Alvarez-Martinez I. . (2024b). Elastic band training versus multicomponent training and group-based dance on morphological variables and physical performance in older women: a randomized controlled trial. Life 14:1362. doi: 10.3390/life14111362, 39598161 PMC11595942

[ref25] Herold F. Hamacher D. Schega L. Müller N. G. (2018). Thinking while moving or moving while thinking—concepts of motor-cognitive training for cognitive performance enhancement. Front. Aging Neurosci. 10:228. doi: 10.3389/fnagi.2018.00228, 30127732 PMC6089337

[ref26] Herold F. Törpel A. Schega L. Müller N. G. (2019). Functional and/or structural brain changes in response to resistance exercises and resistance training lead to cognitive improvements: a systematic review. Eur. Rev. Aging Phys. Act. 16:10. doi: 10.1186/s11556-019-0217-2, 31333805 PMC6617693

[ref27] Iso-Markku P. Aaltonen S. Kujala U. M. Halme H. L. Phipps D. Knittle K. . (2024). Physical activity and cognitive decline among older adults: a systematic review and meta-analysis. JAMA Netw. Open 7:e2354285. doi: 10.1001/jamanetworkopen.2023.54285, 38300618 PMC10835510

[ref28] Kim Y. J. Park H. Park J. H. Park K. W. Lee K. Kim S. . (2020). Effects of multicomponent exercise on cognitive function in elderly Korean individuals. J. Clin. Neurol. 16, 612–623. doi: 10.3988/jcn.2020.16.4.612, 33029968 PMC7541979

[ref29] Lagally K. M. Robertson R. J. (2006). Construct validity of the OMNI resistance exercise scale. J. Strength Cond. Res. 20, 252–256. doi: 10.1519/R-17224.1, 16686549

[ref30] Li A. Sun Y. Li M. Wang D. Ma X. (2024). Effects of elastic band resistance training on the physical and mental health of elderly individuals: a mixed methods systematic review. PLoS One 19:e0303372. doi: 10.1371/journal.pone.0303372, 38739588 PMC11090353

[ref32] Liu-Ambrose T. Nagamatsu L. S. Graf P. Beattie B. L. Ashe M. C. Handy T. C. (2010). Resistance training and executive functions: a 12-month randomized controlled trial. Arch. Intern. Med. 170, 170–178. doi: 10.1001/archinternmed.2009.494, 20101012 PMC3448565

[ref33] Liu-Ambrose T. Nagamatsu L. S. Voss M. W. Khan K. M. Handy T. C. (2012). Resistance training and functional plasticity of the aging brain: a 12-month randomized controlled trial. Neurobiol. Aging 33, 1690–1698. doi: 10.1016/j.neurobiolaging.2011.05.010, 21741129

[ref31] Liu H. Sun Z. Zeng H. Han J. Hu M. Mao D. . (2025). Meta-analysis of the effects of multi-component exercise on cognitive function in older adults with cognitive impairment. Front. Aging Neurosci. 17:1551877. doi: 10.3389/fnagi.2025.1551877, 40297493 PMC12034652

[ref34] Livingston G. Huntley J. Liu K. Y. Costafreda S. G. Selbæk G. Alladi S. . (2024). Dementia prevention, intervention, and care: 2024 report of the lancet standing commission. Lancet 404, 572–628. doi: 10.1016/S0140-6736(24)01296-0, 39096926

[ref35] Luo H. Zheng Z. Yuan Z. Hu H. Sun C. (2024). The effectiveness of multicomponent exercise in older adults with cognitive frailty: a systematic review and meta-analysis. Arch. Public Health 82:229. doi: 10.1186/s13690-024-01441-y, 39614334 PMC11605886

[ref36] Marfell-Jones M. J. Stewart A. D. de Ridder J. H. (2012). International Standards for Anthropometric Assessment. Lower Hutt: International Society for the Advancement of Kinanthropometry.

[ref37] Meng Y. Hu Y. Yang W. Xue Y. Yang S. (2025). Effects of elastic band resistance training on lower limb strength and balance function in older adults: a systematic review and meta-analysis. Front Sports Act Living 7:1649305. doi: 10.3389/fspor.2025.1649305, 41278596 PMC12630110

[ref38] Miller E. K. Cohen J. D. (2001). An integrative theory of prefrontal cortex function. Annu. Rev. Neurosci. 24, 167–202. doi: 10.1146/annurev.neuro.24.1.167, 11283309

[ref39] Ministerio de Salud de Chile. (n.d). Manual de aplicación del Examen de Medicina Preventiva del Adulto Mayor. Santiago: Subsecretaría de Salud Pública, Programa de Salud del Adulto Mayor, División de Prevención y Control de Enfermedades.

[ref40] Nasreddine Z. S. Phillips N. A. Bédirian V. Charbonneau S. Whitehead V. Collin I. . (2005). The Montreal cognitive assessment, MoCA: a brief screening tool for mild cognitive impairment. J. Am. Geriatr. Soc. 53, 695–699. doi: 10.1111/j.1532-5415.2005.53221.x, 15817019

[ref41] Northey J. M. Cherbuin N. Pumpa K. L. Smee D. J. Rattray B. (2018). Exercise interventions for cognitive function in adults older than 50: a systematic review with meta-analysis. Br. J. Sports Med. 52, 154–160. doi: 10.1136/bjsports-2016-096587, 28438770

[ref42] Pesce C. (2012). Shifting the focus from quantitative to qualitative exercise characteristics in exercise and cognition research. J. Sport Exerc. Psychol. 34, 766–786. doi: 10.1123/jsep.34.6.766, 23204358

[ref43] Petro J. L. Ferrari G. Cardozo L. A. Vargas-Molina S. Carbone L. Kreider R. B. . (2025). Validity of rating of perceived exertion scales in relation to movement velocity and exercise intensity during resistance-exercise: a systematic review. Sports Health 17, 621–628. doi: 10.1177/19417381241260412, 38910451 PMC11569527

[ref44] Raichlen D. A. Alexander G. E. (2017). Adaptive capacity: an evolutionary neuroscience model linking exercise, cognition, and brain health. Trends Neurosci. 40, 408–421. doi: 10.1016/j.tins.2017.05.001, 28610948 PMC5926798

[ref45] Reitan R. M. (1958). Validity of the trail making test as an indicator of organic brain damage. Percept. Mot. Skills 8, 271–276. doi: 10.2466/pms.1958.8.3.271

[ref46] Richardson J. T. E. (2011). Eta squared and partial eta squared as measures of effect size in educational research. Educ. Res. Rev. 6, 135–147. doi: 10.1016/j.edurev.2010.12.001

[ref47] Sampaio R. A. C. Sewo Sampaio P. Y. Coelho-Júnior H. J. Onodera C. M. K. Cadore E. L. Gutierrez G. L. . (2020). Elastic band power training improves physical function and health-related quality of life in institutionalized frail older adults. Aging Med. Healthc. 11, 136–141. doi: 10.33879/AMH.114.2020.06018

[ref48] Sánchez-Lastra M. A. Varela S. Cancela J. M. Ayán C. (2022). Upper versus lower body resistance exercise with elastic bands: effects on cognitive and physical function of institutionalized older adults. Eur Geriatr Med. 13, 907–916. doi: 10.1007/s41999-022-00616-6, 35150433 PMC9378322

[ref49] Saragih I. D. Yang Y. P. Saragih I. S. Batubara S. O. Lin C. J. (2022). Effects of resistance bands exercise for frail older adults: a systematic review and meta-analysis of randomised controlled studies. J. Clin. Nurs. 31, 43–61. doi: 10.1111/jocn.15950, 34289511

[ref50] Seidler R. D. Bernard J. A. Burutolu T. B. Fling B. W. Gordon M. T. Gwin J. T. . (2010). Motor control and aging: links to age-related brain structural, functional, and biochemical effects. Neurosci. Biobehav. Rev. 34, 721–733. doi: 10.1016/j.neubiorev.2009.10.005, 19850077 PMC2838968

[ref51] Stillman C. M. Esteban-Cornejo I. Brown B. M. Bender C. M. Erickson K. I. (2020). Effects of exercise on brain and cognition across age groups and health states. Trends Neurosci. 43, 533–543. doi: 10.1016/j.tins.2020.04.010, 32409017 PMC9068803

[ref52] Stuss D. T. Craik F. I. M. (2019). “Alterations in executive functions with aging,” in Cognitive Changes and the Aging brain, eds. Heilman K. M. Nadeau S. E. (Cambridge: Cambridge University Press).

[ref53] Suzuki T. Shimada H. Makizako H. Doi T. Yoshida D. Tsutsumimoto K. . (2013). A randomized controlled trial of multicomponent exercise in older adults with mild cognitive impairment. PLoS One 8:e61483. doi: 10.1371/journal.pone.0061483 PMC3621765 23585901

[ref54] ten Brinke L. F. Bolandzadeh N. Nagamatsu L. S. Hsu C. L. Davis J. C. Miran-Khan K. . (2015). Aerobic exercise increases hippocampal volume in older women with probable mild cognitive impairment: a 6-month randomised controlled trial. Br. J. Sports Med. 49, 248–254. doi: 10.1136/bjsports-2013-093184, 24711660 PMC4508129

[ref55] Tombaugh T. N. (2004). Trail making test a and B: normative data stratified by age and education. Arch. Clin. Neuropsychol. 19, 203–214. doi: 10.1016/S0887-6177(03)00039-8, 15010086

[ref56] United Nations Department of Economic and Social Affairs, Population Division (2022). World Population Prospects 2022: Summary of Results. New York: United Nations.

[ref57] Valdés-Badilla P. Guzmán-Muñoz E. Hernández-Martínez J. Núñez-Espinosa C. Delgado-Floody P. Herrera-Valenzuela T. . (2023). Effectiveness of elastic band training and group-based dance on physical-functional performance in older women with sarcopenia: a pilot study. BMC Public Health 23:2113. doi: 10.1186/s12889-023-17014-7, 37891589 PMC10604857

[ref58] Vásquez-Carrasco E. Albornoz-Gómez V. Cerda-González E. González-Guerra C. Salazar-Díaz J. Hernández-Martínez J. . (2026). Effects of cognitive-motor interventions on serum BDNF levels in adults and older population post-stroke: a systematic review and meta-analysis of randomized controlled trials. Front. Pharmacol. 17:1835358. doi: 10.3389/fphar.2026.1835358, 42181896 PMC13190536

[ref59] Vásquez-Carrasco E. Hernández-Martínez J. Cid-Calfucura I. Guzmán-Muñoz E. Ruiz C. Baeza C. . (2025). Effects of different interventions using taekwondo, boxing, and elastic band training on body composition and physical function in Chilean older women: a randomized controlled trial. Life 15:1049. doi: 10.3390/life15071049, 40724551 PMC12300093

[ref60] Voelcker-Rehage C. Niemann C. (2013). Structural and functional brain changes related to different types of physical activity across the life span. Neurosci. Biobehav. Rev. 37, 2268–2295. doi: 10.1016/j.neubiorev.2013.01.028, 23399048

[ref61] Voss M. W. Vivar C. Kramer A. F. van Praag H. (2013). Bridging animal and human models of exercise-induced brain plasticity. Trends Cogn. Sci. 17, 525–544. doi: 10.1016/j.tics.2013.08.001, 24029446 PMC4565723

[ref9002] Wang Z. Xu X. Yang X. Wang S. S. Zhou Y. Li Y . (2024). Effects of multicomponent exercise on cognitive function in persons with mild cognitive impairment: a systematic review and meta-analysis. Int J Nurs Stud. 158:104843. doi: 10.1016/j.ijnurstu.2024.104843, 39116586.

[ref62] Wollesen B. Voelcker-Rehage C. (2014). Training effects on motor–cognitive dual-task performance in older adults: a systematic review. Eur. Rev. Aging Phys. Act. 11, 5–24. doi: 10.1007/s11556-013-0122-z

[ref9001] World Health Organization. (2020). WHO guidelines on physical activity and sedentary behaviour. Geneva: World Health Organization. 10.1136/bjsports-2020-102955 PMC7719906 33239350

[ref9003] Xu L. Gu H. Cai X. Zhang Y. Hou X. Yu J. . (2023). The effects of exercise for cognitive function in older adults: a systematic review and meta-analysis of randomized controlled trials. Int J Environ Res Public Health. 20:1088. doi: 10.3390/ijerph20021088, 36673844. PMC9858649

